# Global DNA methylation changes and differential gene expression in *Anaplasma phagocytophilum*-infected human neutrophils

**DOI:** 10.1186/s13148-015-0105-1

**Published:** 2015-07-29

**Authors:** Sara H. G. Sinclair, Srinivasan Yegnasubramanian, J. Stephen Dumler

**Affiliations:** Graduate Program in Cellular and Molecular Medicine, The Johns Hopkins University School of Medicine, Baltimore, MD USA; Department of Pathology, The Johns Hopkins University School of Medicine, Baltimore, MD USA; Department of Pathology, University of Maryland School of Medicine, Baltimore, MD USA; Department of Microbiology and Immunology, University School of Medicine, Baltimore, MD USA; Sidney Kimmel Comprehensive Cancer Center, Johns Hopkins University School of Medicine, Baltimore, MD USA

**Keywords:** DNA methylation, *Anaplasma phagocytophilum*, Hypermethylation, Cellular reprogramming, MHC locus

## Abstract

**Background:**

*Anaplasma phagocytophilum* is an obligate intracellular prokaryotic pathogen that both infects and replicates within human neutrophils. The bacterium represses multiple antimicrobial functions while simultaneously increasing proinflammatory functions by reprogramming the neutrophil genome. Previous reports show that many observed phenotypic changes are in part explained by altered gene transcription. We recently identified that large chromosomal regions of the neutrophil genome are differentially expressed during *A. phagocytophilum* infection. Because of this, we sought to determine whether gene expression programs altered by infection were the result of changes in the host neutrophil DNA methylome.

**Results:**

Within 24 h of infection, marked increases in DNA methylation were observed genome-wide as compared with mock-infected controls and pharmacologic inhibition of DNA methyltransferases resulted in decreased bacterial growth. New regions of DNA methylation were enriched at intron and exon junctions; however, intragenic methylation did not correlate with altered gene expression. In contrast, intergenic DNA methylation was associated with *A. phagocytophilum*-induced gene expression changes. Within the major histocompatibility complex locus on chromosome 6, a region with marked changes in infection-induced differential gene expression, new regions of methylation were localized to boundaries of active and inactive chromatin.

**Conclusions:**

These data strongly suggest that *A. phagocytophilum* infection, in addition to altering histone structure, alters DNA methylation and the epigenome of its host cell to promote survival and replication, providing evidence that such bacterial infection can radically alter the epigenome of its host cell.

**Electronic supplementary material:**

The online version of this article (doi:10.1186/s13148-015-0105-1) contains supplementary material, which is available to authorized users.

## Background

*Anaplasma phagocytophilum* is an obligate intracellular prokaryotic pathogen of mammalian neutrophils. Transmitted by *Ixodes* spp. ticks, *A. phagocytophilum* infection results in manifestations ranging from subclinical infection or mild self-limited fever to severe infection requiring hospitalization or causing death [1, 2]. The neutrophil is an unusual host cell for any microorganism, especially bacteria, due to its role in innate immunity where its primary function is to recognize and kill microbes. Despite this inhospitable environment, *A. phagocytophilum* reprograms its host cell so that the bacterium can survive long enough for continued replication. The major phenotypic alterations among infected human neutrophils include complex functions such as decreased antimicrobial activity and respiratory burst, reduced phagocytosis, reduced margination and emigration across the endothelium, delayed apoptosis, and an increased production and release of proinflammatory cytokines, chemokines, and proteases [3–8]. These functional alterations allow the bacterium to survive long enough to replicate and spread to newly recruited host cells and to ensure that its host cell remains viable within the intravascular network until it can be accessed by a feeding tick.

The observed changes in host cell function are partially explained by alterations in granulocyte gene transcription [9–11]. Decreased respiratory burst allows for prolonged inhibition of superoxide production favoring bacterial survival and likely results from the downregulation of two key components of the NADPH oxidase required for proper oxidase assembly: *CYBB*, which encodes gp91^*phox*^, and *RAC2*, a GTPase [3, 12]. Likewise, transcription of *BCL2* family member genes are maintained during *A. phagocytophilum* infection which results in delayed neutrophil apoptosis [7, 13] and increased transcript levels of cytokines and chemokines such as IL-1α and IL-8 contribute to exaggerated inflammatory responses that recruit new neutrophil hosts [14–16].

Precisely how *A. phagocytophilum* coordinates the transcriptional changes that allow it to reprogram host cell functions is not known but is unlikely to result from an accumulation of individual signaling pathways targeted by unique pathogen protein effectors. We described decreased histone acetylation and methylation at multiple defense gene promoters with infection [17] and enhanced intracellular bacterial growth with *HDAC-1* and −2 expression and activity, as well as the dose-dependent decrease in bacterial load effects of *HDAC1* silencing and HDAC pharmacologic inhibition [17]. We also showed that transfection of the nucleomodulin and type IV system secretion substrate AnkA of *A. phagocytophilum* alone mimics many transcriptional changes observed with infection by directly binding host cell DNA in multiple genomic regions, including at the *CYBB* proximal promoter where it decreases histone H3 acetylation and gene expression [18]. Previously, we showed that the *A. phagocytophilum* nucleomodulin AnkA recruits HDAC1 to the *CYBB* promoter, and the altered chromatin configuration excludes RNA polymerase II recruitment thereby silencing expression [18]. These observations prove an epigenetic basis for silencing at *CYBB* and suggest that *A. phagocytophilum* infection alters transcriptional programs of its host cells via genome-wide epigenetic alterations.

HDACs target nucleosomal histone proteins to create modifications that alter gene promoter accessibility to transcriptional regulators. Interestingly, HDACs also complex with DNA methyl binding proteins (MBDs) and work to coordinately alter transcriptional programs. DNA methylation at CpG dinucleotides is an important epigenetic regulator of cellular differentiation, neoplasia, and metastasis [19–22]. Moreover, expression of DNA methyltransferase 3A (DNMT3A), a key enzyme for de novo DNA methylation, is upregulated in *A. phagocytophilum*-infected neutrophils ex vivo [9]. Given the complex interplay between these two epigenetic modifiers and the induction of histone deacetylation by *A. phagocytophilum*, we hypothesized that *A. phagocytophilum* also alters the DNA methylome of human neutrophils that in part determines transcriptional reprogramming.

Precedence for bacterial influence on host DNA methylation patterns has been established in both *Helicobacter pylori* and uropathogenic *Escherichia coli* (UPEC) infections [23]. Gastric mucosa infected with *H. pylori* shows increased DNA methylation patterns at CpG islands but is hypothesized to be a result of infection-related inflammation rather than bacterial-induced DNA methylation (Na and Woo, 2014). It is important to note that *H. pylori* is generally considered to be an extracellular organism so its effects on gastric mucosa are not due to direct invasion and replication inside the stomach tissue. UPEC, which does actively invade uroepithelial cells, was shown to directly influence the methylation of target genes, but its genome-wide effects have not yet been elucidated [24]. Here, we found that *A. phagocytophilum* induces hypermethylation of the human neutrophil genome within 24 h of infection. Although several bacteria are implicated in alterations of host cell DNA methylation [23], this is the first demonstration that infection by an intracellular bacterium alters host DNA methylation patterns on a genome-wide scale. The data strongly suggest that this is part of a coordinated reprogramming of host cell functions which leads to improved microbial fitness by promoting intracellular survival, replication, and spread.

## Results

### *A. phagocytophilum* induces genome-wide hypermethylation

**Fig. 1:**
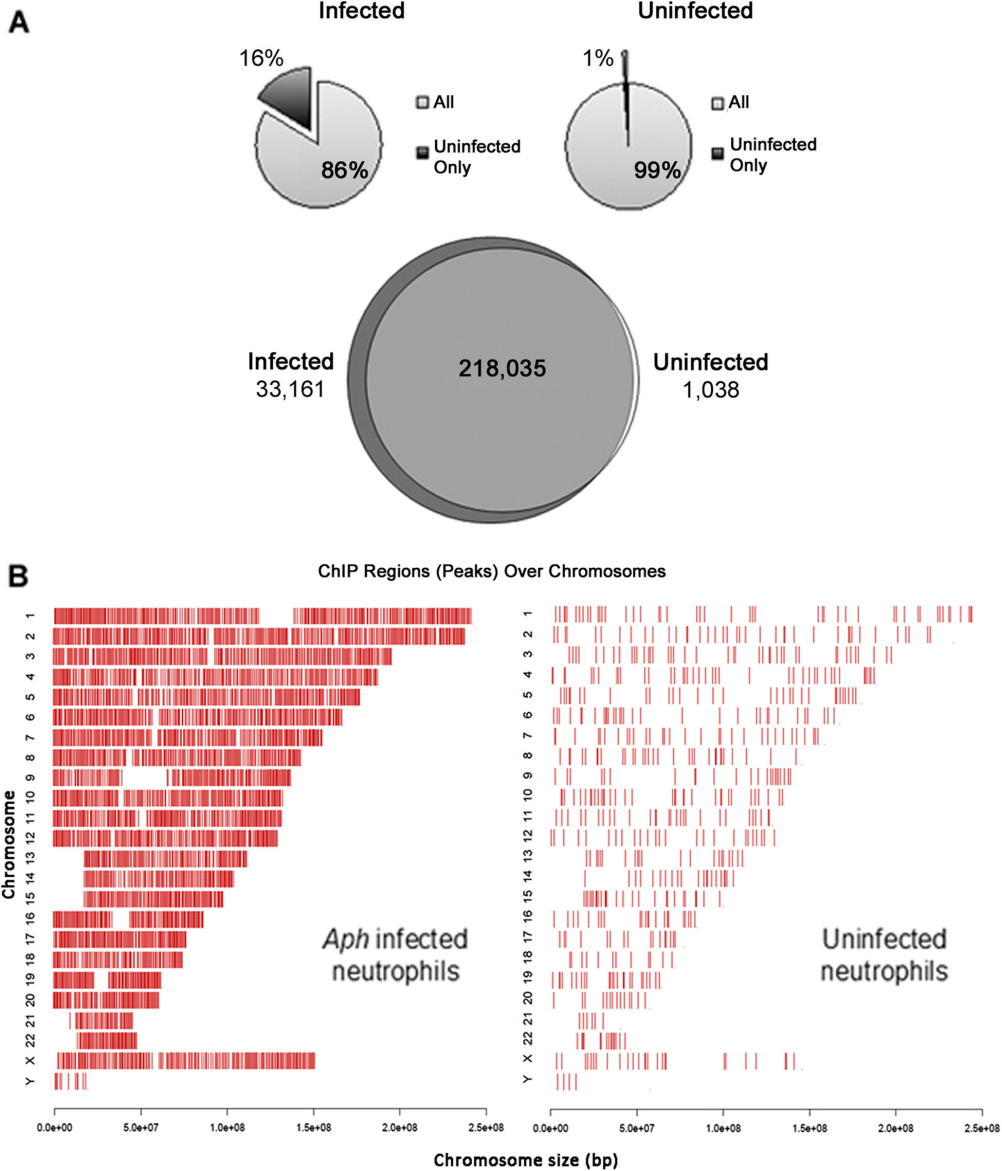
*Anaplasma phagocytophilum* induces genome-wide changes in DNA methylation of human peripheral blood neutrophils. The quantity of new regions of DNA methylation was compared between infected and uninfected samples. **a** Pie charts demonstrate the proportion of new methylation peaks among all meDNA that mapped to infected cells only (*left dark wedge*) versus uninfected cells (*right dark narrow wedge*). While a large percentage of peaks are shared between samples, infected samples have more new regions of methylation as shown by the Venn diagram. The number of methylated regions shown is the average of the three donors. **b** CEAS was used to linearly map chromosomal locations of all methylated peaks unique to infected or uninfected samples. Representative image of a single donor where regions of DNA methylation (*bars*) unique to *A. phagocytophilum*-infected neutrophils and to uninfected human neutrophils are shown per chromosome, by their linear location on the x-axis. *Each bar* represents a new region of methylation found only in its respective sample. Roughly 30,000 new regions of methylation are unique to infected samples and cover the entire genome. In comparison, the uninfected samples show an equally diverse distribution of ~1000 newly methylated regions

Human peripheral blood neutrophils were isolated and infected with *A. phagocytophilum* for 24 h before DNA isolation. We used methylated DNA binding domain (MBD) enrichment and next generation sequencing approach (MBD-seq) to analyze patterns of DNA methylation across the whole genome of *A. phagocytophilum*-infected and uninfected primary human neutrophils. Regions significantly enriched for DNA methylation were determined using either Model-based analysis of ChIP-Seq software (MACS) [25] or Statistical model for Identification of ChIP-Enriched Regions software (SICER) [26] using stringency cutoffs of *p* = 10e-6 (MACS) or/and FDR = 0.01 (SICER) with a minimum length of 200 bp. MACS peaks are used for all figures unless otherwise noted. Using peaks called by MACS, the majority of methylated regions (~84 %) across the genome were maintained between uninfected and infected neutrophils; there were a significant number of newly methylated regions (~30,000 or 16 %) in the infected neutrophils as compared to uninfected samples (Fig. 1a). Regions of methylation unique to infected or uninfected samples were mapped linearly along chromosomes, demonstrating that the changes in DNA methylation associated with infection were pervasive across all chromosomes (Fig. 1b) and confirmed that alterations in DNA methylation patterns occurred on a genome-wide scale. Luminometric methylation assay (LUMA) analysis of the same DNA samples further confirmed an increase in the percentage of DNA methylation with *A. phagocytophilum* infection of human neutrophils.

### Inhibition of DNMTs with 5-azacytidine slows *A. phagocytophilum* growth

**Fig. 2:**
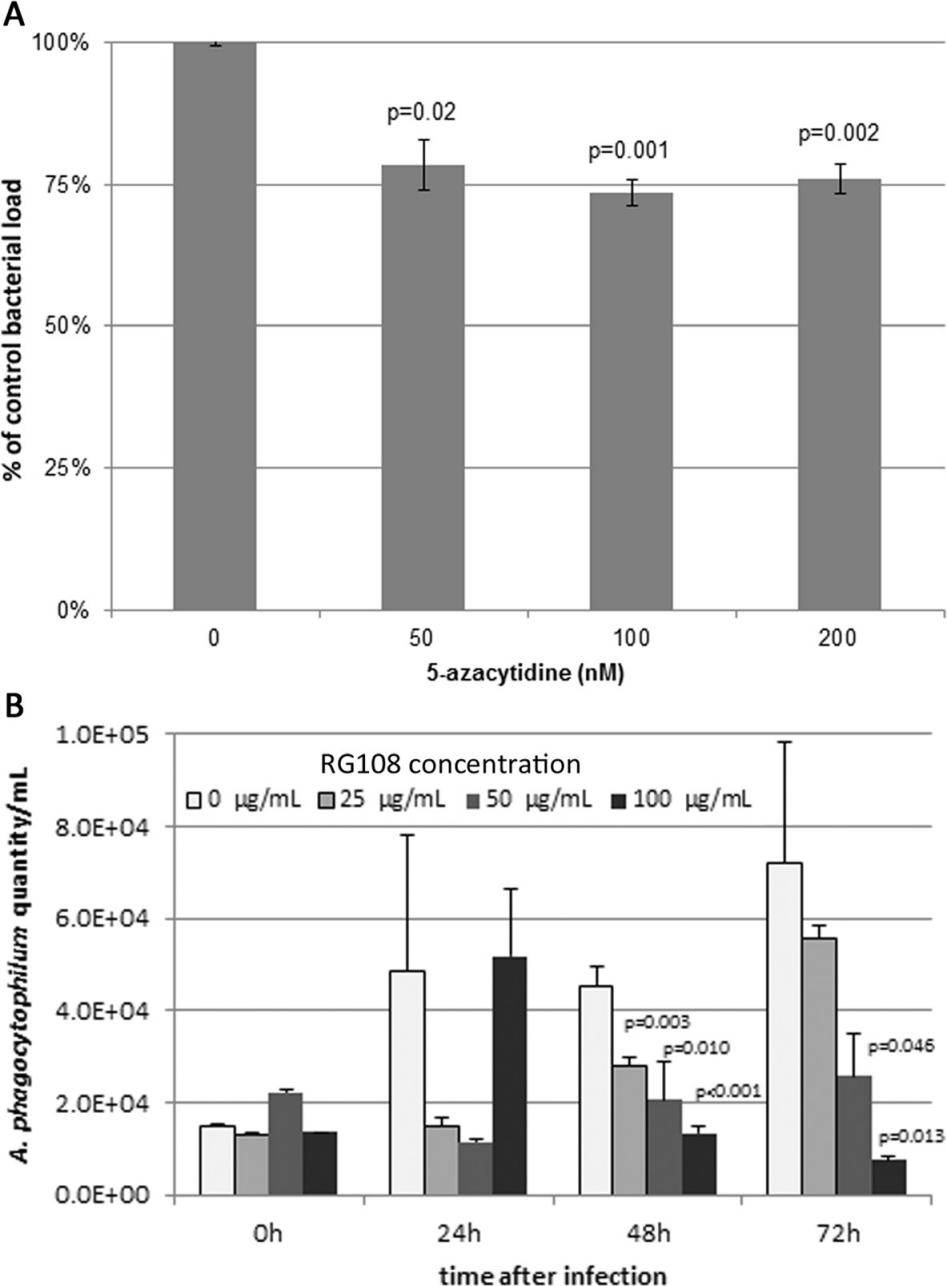
DNA methyltransferase inhibition abrogates *A. phagocytophilum* growth in HL-60 cells. **a** Treatment for 24 h with the DNMT inhibitor 5-azacytidine 24 h post *A. phagocytophilum* infection results in a reduction in bacterial load regardless of inhibitor concentration. The bacterial load in three replicates was determined using a qRT PCR assay of *A. phagocytophilum msp2/p44* normalized to HL-60 cell *ACTB* and to vehicle treated cells. **b** Treatment with the DNMT inhibitor RG108 for 72 h post *A. phagocytophilum* infection results in a dose-dependent reduction in bacterial load at 48 and 72 h. Results are normalized to HL-60 cell *ACTB*, but displayed in comparison with vehicle-only-treated cells. *P* values displayed above the bars were calculated by two-sided unequal variance Student’s *t* tests by comparison with vehicle only-treated samples at the same time interval after infection

In concert with increased global DNA methylation, transcription of *DNMT3A* is upregulated with *A. phagocytophilum* infection of human neutrophils ex vivo [9]. To determine whether methylation of host cell DNA is essential for bacterial survival and growth, we investigated the pharmacologic effects of two DNA methyltransferase (DNMT) inhibitors with distinct mechanisms of action, 5-azacytidine and RG108, on *A. phagocytophilum* infection using 24 h (5-AZA) or 24, 48, and 72 h (RG108) of infection. All transretinoic acid (ATRA)-differentiated HL-60 promyelocytic cells, commonly used as a cell model for *A. phagocytophilum* infection, were infected with cell-free *A. phagocytophilum* for 24 h before treatment with 5-azacytidine. For RG108 experiments, cultures of (ATRA)-differentiated HL-60 cells were adjusted to 20 % infected cells before treatment. After infection or addition of the inhibitor, DNA from three individually infected and 5-AZA treated cultures was extracted and bacterial load was determined using a quantitative real time PCR assay comparing *A. phagocytophilum msp2/p44* normalized to a human *ACTB* standard. Bacterial replication was ~25 % less than the DMSO vehicle control with all concentrations of 5-AZA (Fig. 2a). For RG108, infection in the vehicle only-treated cultures did not alter normal bacterial growth, with propagation of 5-fold by 72 h (Fig. 2b); however, treatment with all doses of RG108 resulted in reduced dose-dependent bacterial growth at 48 and 72 h posttreatment. Growth was also reduced in most treated cultures at 24 h, but the changes were not statistically significant. Treatment with RG108 resulted in a net reduction in 5-methyl cytosine content with most drug doses over 3 days of treatment as compared with no drug treatment, suggesting that lack of off-target drug effects as an alternate explanation for decreased *A. phagocytophilum* growth (Additional file 1: Figure S1).

### Characterization of DNA methylation across common gene features

**Fig. 3:**
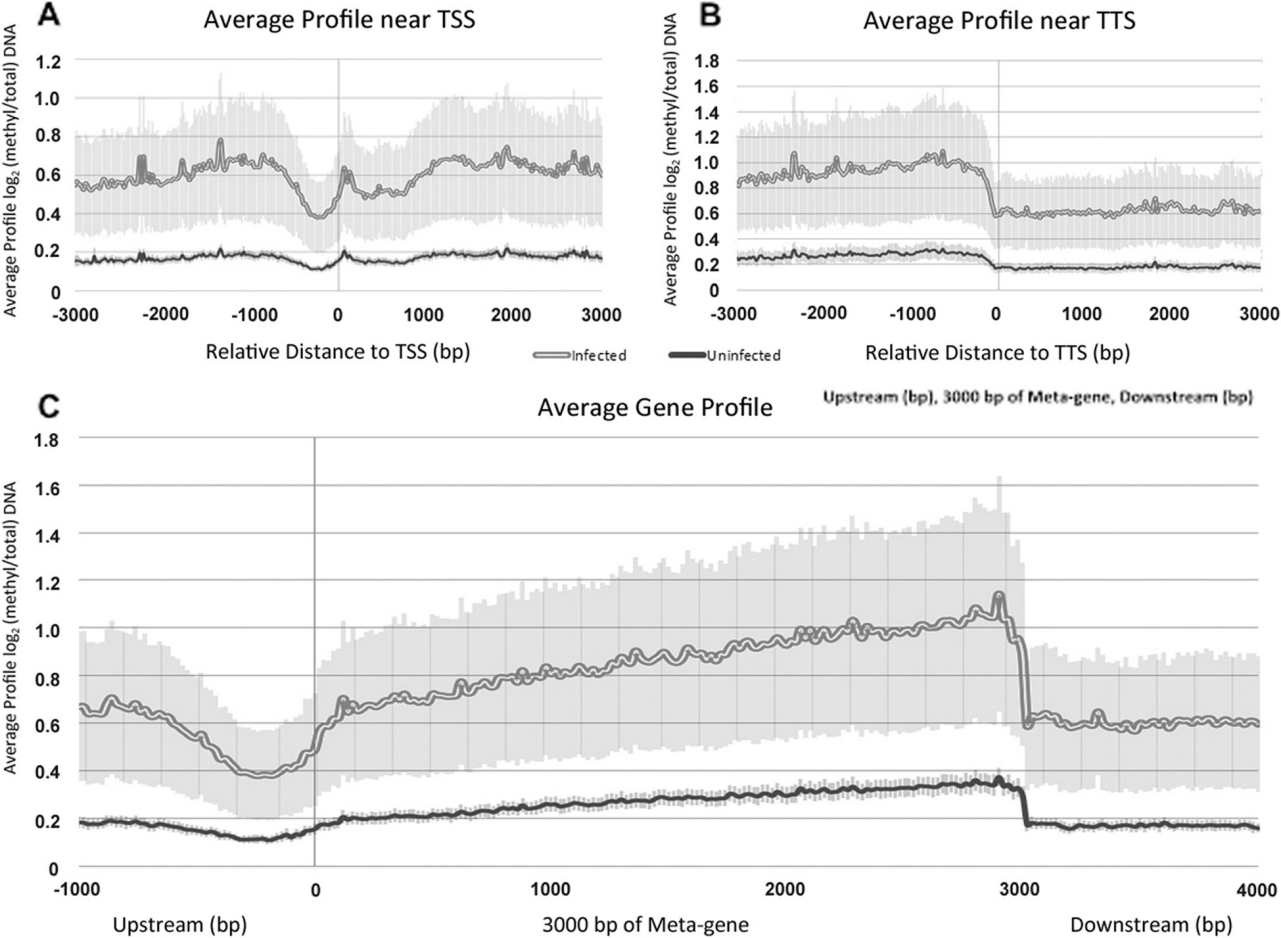
Average signal profile for 5mC enrichment. **a** Transcriptional start sites (TSS), **b** transcription termination sites (TTS), and **c** across an average gene was calculated using CEAS. *Light gray* represents the average profile of enrichment for *A. phagocytophilum*-infected peripheral blood neutrophils, and *dark gray* for uninfected for all three donors. *Shaded zones* show the standard error of the mean for each donor

We next sought to determine which gene features were most prominently methylated throughout the genome with infection. The average methylation signal surrounding all transcription start sites (TSS), termination sites (TTS), and across genes for each individual donor is shown in Fig. 3. Both *A. phagocytophilum*-infected and uninfected samples showed similar relative profiles of DNA methylation across gene features. However, compared to uninfected, the profiles from *A. phagocytophilum*-infected samples showed a more pronounced decrease in DNA methylation immediately upstream of the TSS (Fig. 3a), whereas downstream of the TSS, DNA methylation rose sharply and continued to increase throughout the gene body until the TTS (Fig. 3b, c). These differences were persistent even after adjusting for differences in the absolute numbers of sequencing reads.

**Fig. 4:**
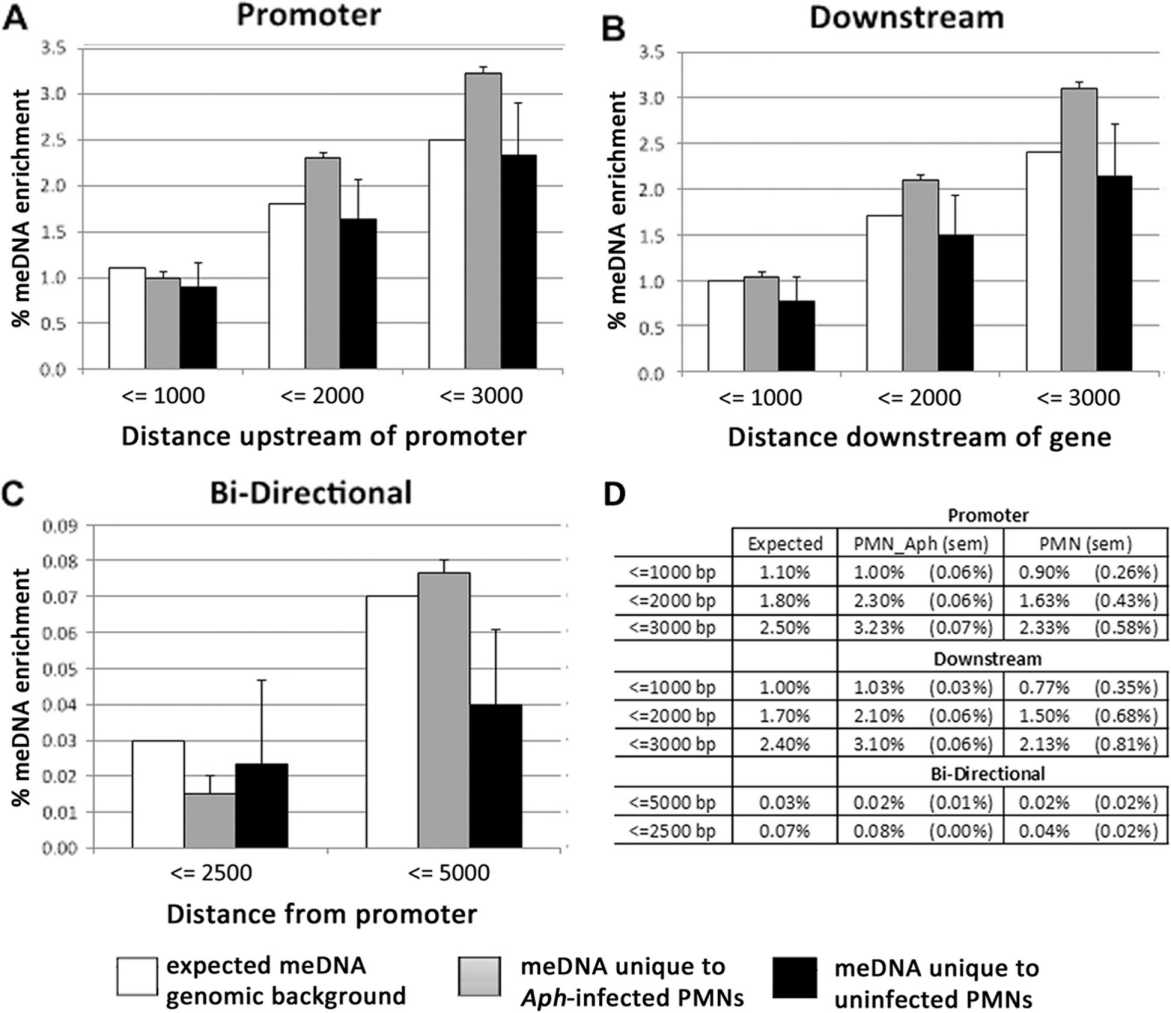
Infection of human neutrophils with *A. phagocytophilum* increases DNA methylation upstream of promoters and far downstream of transcription termination sites. New regions of DNA methylation enrichment were investigated using CEAS to determine the percentage that were present in **a** promoters, **b** downstream of genes, and **c** in bi-directional promoters. Only the most significant peaks, those with a −10*log_10_ (*p* value) > 100, were analyzed. *White bars* denote the percentage of the genome annotated to that particular feature (genomic background), *gray bars* denote average percentage of regions of DNA methylation unique to *A. phagocytophilum*-infected human peripheral blood neutrophils (PMN.Aph), and *black bars* denote the average percentage of regions of methylation unique to uninfected cells (PMN); error bars are standard error of the mean (sem). Average values are calculated using all three donors. Tables in **d** show the exact percentage of DNA methylation enrichment for each particular feature where PMN.Aph represents regions unique to infected cells, PMN represents regions unique to uninfected cells, and sem for each group

**Fig. 5:**
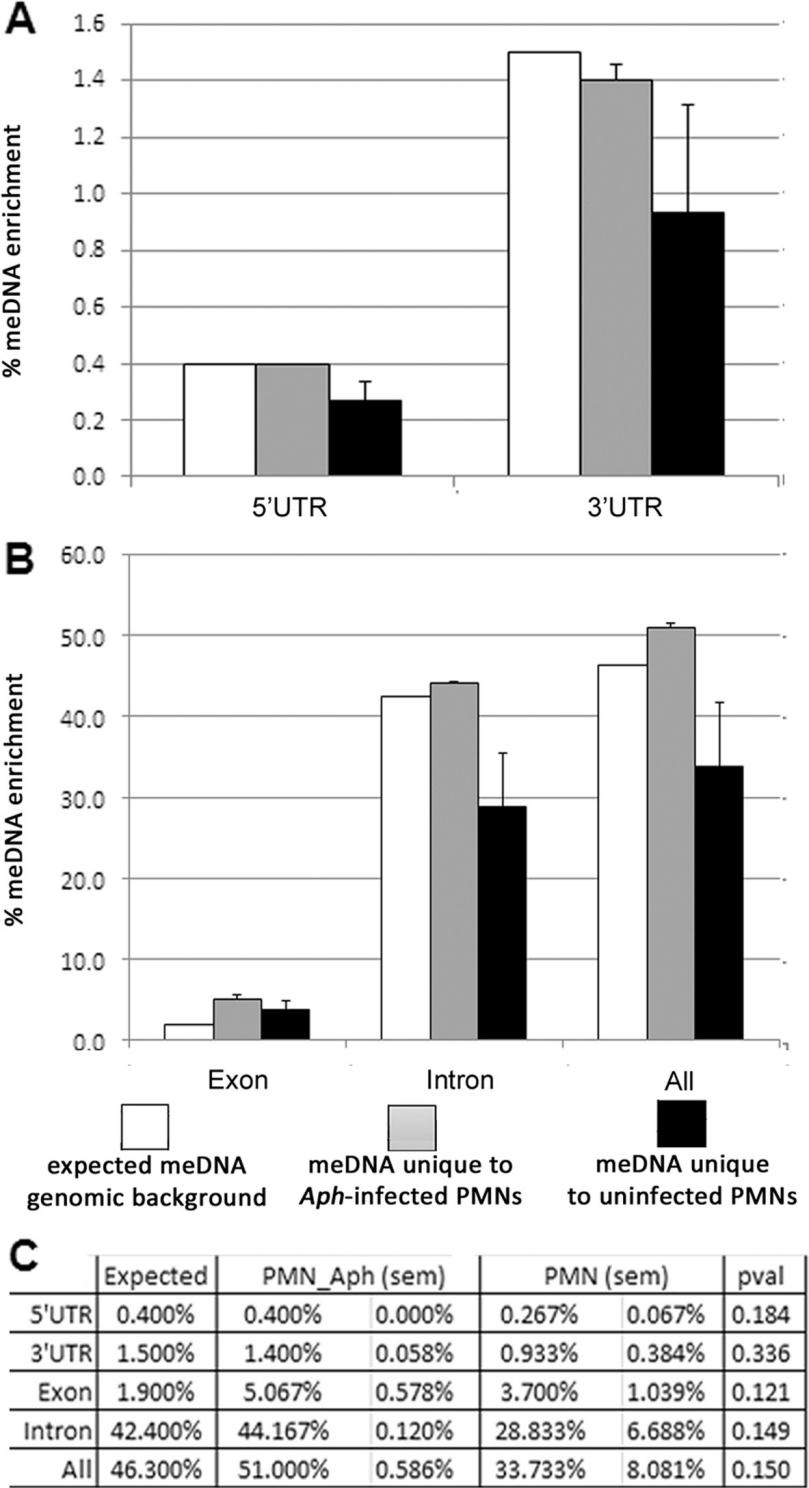
*A. phagocytophilum* infection causes an increase in exon DNA methylation. Identification of gene features with DNA methylation enrichment unique to *A. phagocytophilum*-infected neutrophils and uninfected neutrophils was performed using CEAS. Only the most significant peaks, those with a −10*log_10_ (*p* value) > 100, were analyzed. Features investigated include **a** 5′ and 3′ UTRs and **b** exons, introns, and the sum of all gene features. *White bars* denote the percentage of the genome annotated to that particular feature (genomic background), *gray bars* denote the average percentage of regions of DNA methylation unique to *A. phagocytophilum*-infected human peripheral blood neutrophils (PMN.Aph), and *black bars* denote the average percentage of regions of methylation unique to uninfected cells (PMN); error bars show sem for each group. Tables in **c** shows the exact percentage of DNA methylation enrichment for each particular feature and sem for each group. Data shown represent the average of three donors

Because infection with *A. phagocytophilum* induced greater spread and degrees of DNA methylation compared to uninfected cells, we investigated whether the newly methylated regions localized to specific gene features. Regions of methylation were highly associated with genes in *A. phagocytophilum*-infected neutrophils and uninfected neutrophils. Nearly 62 and 66 %, respectively, mapped to or within 3 kb of annotated genes. Infected samples had higher than expected methylation enrichment within 2–3 kb upstream or downstream of genes (Fig. 4) as well as increased methylation of exons (Fig. 5). “Expected” refers to how many reads would be expected to align with each gene feature if there was no DNA methylation enrichment and is used to demonstrate genomic background. Expected values were determined by the cis-regulatory element annotation system (CEAS) software. Nearly 5 % of all newly methylated regions in *A. phagocytophilum*-infected cells fell within exons compared to the expected value of 2 % (Fig. 5). Taken as a whole, new DNA methylation was significantly higher after infection for every gene feature except bidirectional promoters ≤5000 bp from the TSS (*p* = 0.002; Wilcoxon Signed Rank test). However, when examined at each genomic feature, the proportion of new DNA methylation was not significantly different between infected and uninfected neutrophils likely because of a high degree of variation among uninfected neutrophils (Figs. 4 and 5; average coefficient of variation (CV) (0.387) despite minimal variation after infection (average CV 0.056).

**Fig. 6:**
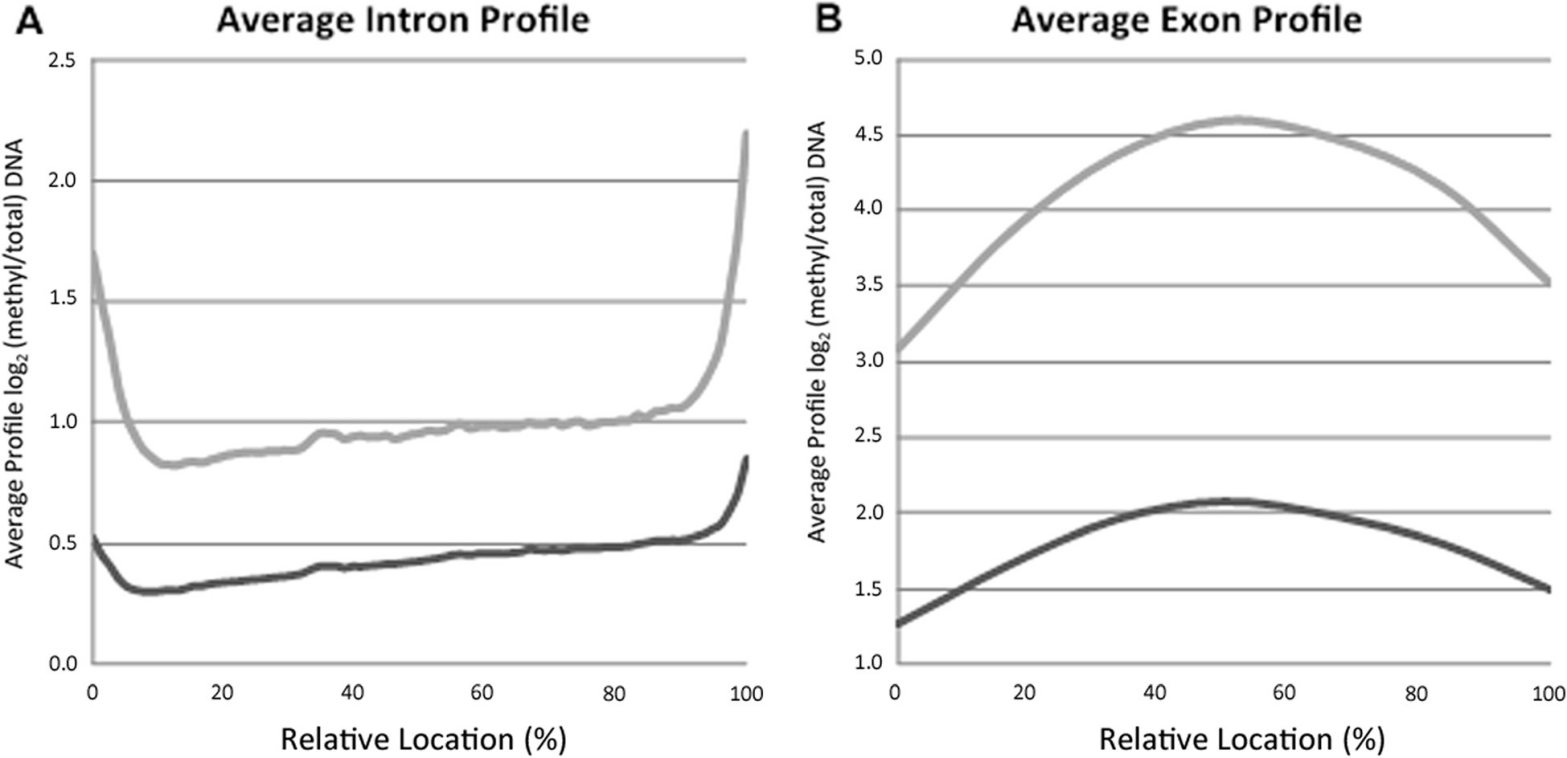
Average signal profile for introns and exons. The average signal profiles for **a** medium-sized introns (842–2715 bp) and **b** exons (109–164 bp) were calculated using CEAS. *Light gray* represents the average enrichment profile for *A. phagocytophilum*-infected human peripheral blood neutrophils, and *dark gray* represents uninfected neutrophils. Averages calculated using all three donors

Intron and exon methylation was averaged, scaled as a fraction between 0 to 100 %, and plotted by position. Because exon and intron lengths vary highly, the default CEAS analysis groups these into three classes by length to calculate average profiles that in turn avoids graphical artifacts due to length-normalization. The profile for all introns had regions of methylation localizing toward the intron/exon junctions (Fig. 6a). For all exons, there was a gradual accumulation of methylation signals toward the middle of the exons (Fig. 6b). The pattern of both profiles suggests that exons could be preferentially methylated and regions of methylation could overlap at intron/exon junctions. Overall, *A. phagocytophilum* infection-induced hypermethylation of the host genome leads to regions of enrichment most often associated with gene bodies or within 3 kb of annotated gene features.

### DNA methylation and transcriptional alterations in the *A. phagocytophilum*-infected neutrophil

We then sought to determine whether or not DNA methylation influenced transcription of the most differentially expressed genes. Genes that were up- or downregulated with a fold-change of 3–6 or >6 standard deviations (SD) from the mean fold-change were assessed for DNA methylation peaks. Since DNA methylation is found to be correlated across genomic regions at a scale of <2 kb [27], if a region of DNA methylation was located within the gene body or ±3 kb from TSS or TTS, it was considered associated with that gene. Using these criteria, we compared the fold-change in expression of 516 genes associated with DNA methylation to the remaining 404 genes not associated with DNA methylation. The average log_2_ differential gene expression (DGE) of 361 methylated genes with between 3–6 SD from the mean was −0.037, and no statistical association with DNA methylation could be identified when compared with 269 genes lacking DNA methylation (*p* = 0.565). At this level, 120 of 361 genes with DNA methylation were upregulated and 100 of 269 genes lacking DNA methylation were upregulated. In contrast, of the 155 methylated genes that were differentially expressed ≥6 SD above the mean, 86 were upregulated (log_2_ DGE 0.694) rather than downregulated, although the resulting association with DNA methylation was not significant compared to 135 non-methylated genes with the same differential gene expression. This is consistent with a previously published report by Borjesson et al. that the most differentially expressed genes induced by *A. phagocytophilum* infection were upregulated [9].

**Fig. 7:**
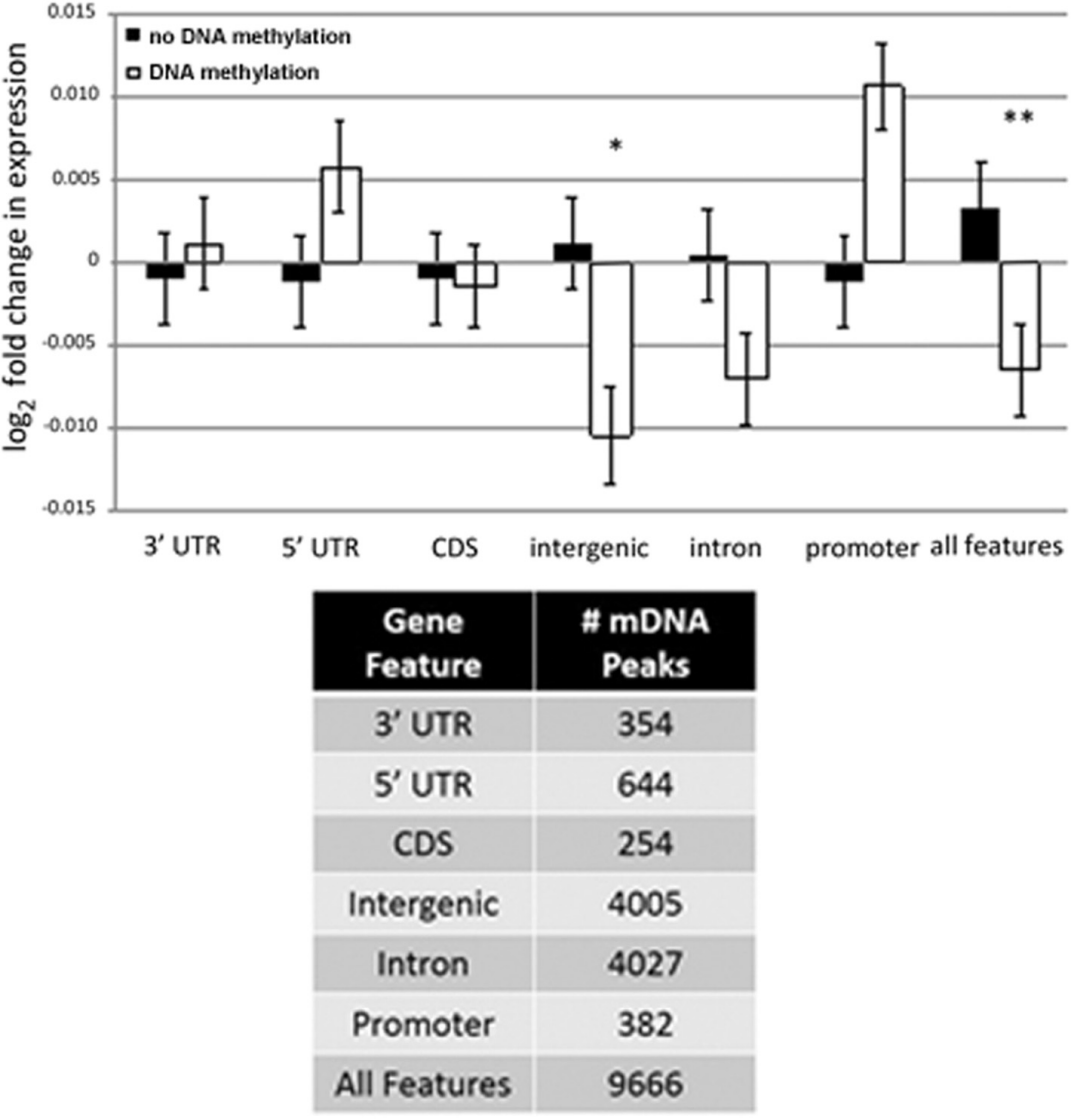
Effect of DNA methylation on gene regulation. Gene features associated with newly methylated regions that also had differential gene expression >3 SD from the mean were sorted by methylation in 3′UTR, 5′UTR, coding sequences (CDS), intergenic regions, introns, and promoters. These were then correlated with log_2_-fold-change (differential gene expression) between infected and uninfected samples for their respective genes. DNA methylation peaks were considered intergenic when located 3 kb or more from a TSS or TTS and effects on transcription were determined for the nearest gene. Transcriptional changes of genes with DNA methylation were compared to those without. *p < 0.05, **p < 0.01. Data are derived from the average of three donors

Next, we investigated whether DNA methylation affects transcription at specific locations of DNA methylation within gene features. Genes associated with DNA methylation were sorted based on the gene feature location of the methylation site. Gene features investigated included both 3′ and 5′ untranslated regions (UTRs), coding sequences (CDS), intronic regions, intergenic regions (>3 kb from TSS or TTS), and promoters (±3 kb from TSS or TTS). Additionally, the fold expression change in genes associated with DNA methylation was compared to those without. Our findings indicate that intergenic DNA methylation had a statistically significant impact on nearby gene expression (*p* < 0.05), but the overall expression fold-change was low (Fig. 7). Genes associated with DNA methylation in any location were generally downregulated (*p* < 0.01). Although statistically significant, it is difficult to know whether these small fold-changes are biologically relevant. All other regions were not significantly associated with DNA methylation, and overall fold-changes were minimal.

Although *A. phagocytophilum* infection induced genome hypermethylation, the native level of genome methylation in neutrophils was high. Paired with the relatively low fold-changes in gene expression and the small proportion of all genes that were differentially regulated, these data could mask correlations that would provide evidence for the effects that *A. phagocytophilum* has on DNA methylation that in turn lead to altered gene expression. Because of this, we set out to identify local effects at the chromosomal level especially given that large regions of chromosomes are regulated similarly during infection [28].

### Correlation of individual new meDNA and differential gene transcription at the MHC locus

The major histocompatibility complex (MHC) locus on chromosome 6 was shown to have local clustering of increased gene expression across a large chromosomal area spanning nearly 4 Mb [28]. This locus also has well-described epigenetic regulation; thus, we focused our attention on the association of gene expression and DNA methylation here. To examine whether increased DNA methylation at the MHC locus on chromosome 6 was related to the observed cluster of differentially regulated genes, we examined newly methylated DNA (meDNA) fold-enrichment at individual genes and created a sliding scale window for both the log_2_ fold-change in expression and newly methylated sites in those windows.

On chromosome 6, there were 687 unique newly methylated genomic sites for which differential gene expression data was also obtained, including 53 unique newly methylated sites in the MHC locus and 22 unique newly methylated sites in the position immediately downstream of MHC that we call “PROX”. When individual newly methylated sites across the entire chromosome were examined, no correlation between individual differential gene expression and meDNA fold-enrichment (ρ −0.0391, *p* = 0.306) or –log_10_*p* value (ρ −0.0174 and *p* = 0.649) could be identified. Although correlation between individual differential gene expression with enrichment of individual newly methylated sites over the MHC locus was higher (ρ 0.2664, *p* = 0.053 and –log_10_*p* value ρ 0.2311; *p* = 0.096), it did not reach a level of significance we defined. Likewise, there was no significant correlation at the downstream control PROX region (meDNA fold-enrichment ρ 0.0864, *p* = 0.702, −log_10_*p* value ρ −0.0212 and *p* = 0.925).

### Correlation of new meDNA enrichment with differential gene transcription over long contiguous linear regions at the MHC locus and chromosome 6

**Fig. 8:**
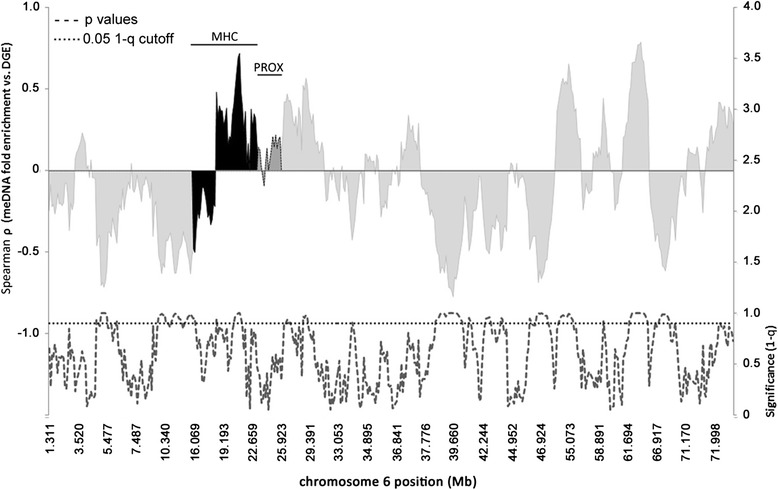
Large genomic regions on chromosome 6 demonstrate correlations between new DNA methylation fold-enrichment and differential gene expression after *A. phagocytophilum* infection of human peripheral blood neutrophils. Chromosome 6 positions are displayed in Mb across the x-axis and the Spearman correlation coefficient (ρ) between fold-enrichment of newly meDNA and log_2_ fold-change in differential gene expression (DGE) is plotted on the left y-axis. *Light gray zones* correspond to regions of chromosome 6 that are upregulated (positive) and downregulated (negative), *black zones* delineate the boundaries of the MHC locus, and the *dark gray zone* depicts the PROX region downstream of the MHC locus. Note that the x-axis is scaled to gene feature density and not linear position on the chromosome. The *lower hatched line plot* corresponds to the right y-axis and shows 1-(*q* value) for the Spearman correlation at the window centered at that genomic position such that peaks correspond to low *q* values. The *dotted line* demarcates where the 1-*q* value is above 0.90 which is equivalent to a *q* value <0.10. MeDNA data are from the average of three donors. Expression data are derived from GSE2405 [9]

Gene expression is often markedly influenced by meDNA and histone chromatin, leading to changes in three-dimensional organization of chromatin itself [29, 30]. To determine whether specific genomic loci over Mb ranges on chromosomes were significantly enriched for newly methylated DNA in areas where a correlation with differential gene expression could be demonstrated, we plotted ρ (rho), the Spearman correlation coefficient for fold-enrichment of new meDNA, and differential gene expression over windows (median 4.2 Mb (IQR 3.9); median 8 unique meDNA features (IQR 2)) against the physical chromosome position of each window on the p and q arms of chromosome 6. Figure 8 demonstrates the average positive or negative correlations between windows of newly methylated DNA and DGE expression in linearly contiguous chromosomal regions that are likely to interact in three dimensions over the chromosome territories. We also plotted the *q* value (1-q) for the correlations at each window to better visualize genomic regions where DNA methylation and DGE were significantly correlated and where there could be a broad impact on gene transcription. The analysis demonstrated long genomic stretches of positive and negative correlation between fold-enrichment in new DNA methylation and differential gene expression in the windows, including several at which significant associations were found, such as at the MHC locus over 88 Kb. Altogether, there were 11 long linear regions on chromosome 6 for which new DNA methylation was significantly correlated (*q* value <0.10); 6 regions comprising 27.5 Mb (16 % of chromosome 6) were negatively correlated, whereas 5 regions comprising 12.1 Mb (7 % of chr6) were positively correlated. To explore these long range genomic associations between new DNA methylation and differential gene expression, we examined these large scale windows on chromosome 6 and the MHC locus in more detail.

**Fig. 9:**
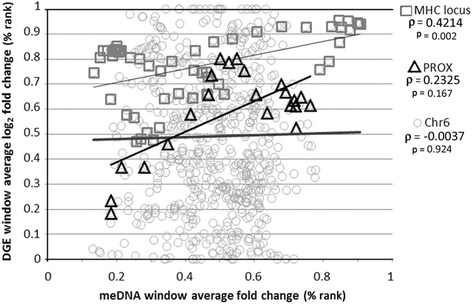
Changes in meDNA with *A. phagocytophilum* infection correlate with regions of genomic differential expression. Increased DNA methylation is significantly correlated with differential gene expression across the MHC locus on chromosome 6 but not over the entire chromosome (Chr6) or across a similarly-sized control PROX genomic region immediately downstream of the MHC locus. MeDNA data are from the average of three donors. Expression data are derived from GSE2405 [9]

Across chromosome 6, as observed when individual differential gene expression and newly meDNA were examined, there was no correlation (*ρ* = −0.0037; *p* = 0.924) between windows with newly methylated DNA fold-enrichment and log_2_-fold differential gene expression windows (median window size 3.9 Mb (IQR 3.6)). In contrast, across the 3.1 Mb MHC region analyzed, there was a strongly positive correlation between window average new meDNA fold-increase and window average log_2_-fold-change for differential expression (*ρ* = 0.422; *p* = 0.002); for the similar sized (3.2 Mb) region immediately downstream from the MHC locus (PROX), the correlation was not significant (*ρ* = 0.233; *p* = 0.310) (Fig. 9). When expression was sorted by upregulated vs. downregulated windows over the MHC locus, a significant positive correlation was observed for upregulated (*ρ* = 0.447; *p* = 0.003) but not downregulated conditions (*ρ* = 0.133; *p* = 0.683). Of interest, when windows were sorted by intragenic vs. intergenic features, there was a significant association of new meDNA vs. differential regulation at the MHC locus for intragenic (*ρ* = 0.644; *p* = 0.001; 22 unique sites) but not for intergenic features (*ρ* = 0.144; *p* = 0.438; 31 unique sites), but the converse was true at PROX (intergenic *ρ* = 0.788; *p* = 0.002; 10 unique sites; intragenic *ρ* = 0.042; *p* = 0.919; 12 unique sites) (Additional file 2: Figure S2).

To deconstruct this further, we subdivided individual DNA methylation peaks and differentially expressed genes over chromosome 6 into their gene regions: intron, CDS, intergenic, and promoter. Here, no single gene region of chromosome 6 had new DNA methylation fold-enrichment that significantly correlated, either positively or negatively, with differential gene transcription (Spearman *p* ≥ 0.091), although the number of meDNA peaks in many categories was relatively small. Only 16 peaks for meDNA enrichment were found in 9 distinct gene promoters (*BMP6*, *NOL7*, *FRS3*, *PTK7*, *RCAN2*, *GJA1*, *SYNJ2*, *TULP4*, and *PLG*), yielding a Spearman *ρ* value of −0.438 (*p* = 0.091). Because those peaks were located outside of gene bodies, they were grouped with intergenic regions. This grouping increased the intergenic region Spearman coefficient to −0.017, suggesting that although these 16 regions could influence expression of their respective genes, the likely impact across all genes on chromosome 6 is not high. There was insufficient data information about whether and how DNA methylation at other genomic regions affects gene expression since similar findings were obtained when the windows corresponding to these regions were evaluated. Here, the greatest correlation was in the 5′ UTR regions, yet the *ρ* value was −0.223, with a *p* value of 0.247. The window regions corresponding to the MHC locus could not be evaluated in the same manner because of the paucity of data over windows in this region. Yet in specific genomic loci, the impact of newly meDNA regions is important, especially considering that newly meDNA was present in 47 % of intergenic and promoter regions across chromosome 6 but in 58 % of the intergenic regions in the MHC locus.

## Discussion

Alteration of DNA methylation patterns is well documented in stem cell differentiation, cellular reprogramming, cancers, and other disease states but not in bacterial infections [21, 22, 31–34]. Previously, several groups suggested that infection by microbial pathogens induces hypermethylation of candidate genes involved in oncogenesis [35, 36, 24]. *Helicobacter pylori* infection causes chronic inflammation of surrounding gastric tissue resulting in aberrant DNA methylation patterns which can contribute to neoplastic transformation [36]. Uropathogenic *E. coli* can induce hypermethylation of *CDKN2A*, a cyclin-dependent kinase inhibitor and tumor suppressor gene, leading to increased proliferation of the urothelium [24]. Most recently, methylomes of ocular adnexal extranodal marginal zone B-cell lymphoma (EMZL) tumors from patients with *Chlamydophila psittaci* infection were compared to EMZL *C. psittaci*-negative tumors [37]. Differentially methylated regions of infected samples hierarchically clustered together suggesting changes in DNA methylation patterns were specific to *C. psittaci* infection [37]. Though informative, two of these studies investigated tissue samples which inevitably contain heterogeneous cell populations, and the other only investigated several candidate genes. Here, we show a direct link between infection of *A. phagocytophilum* and genome-wide alterations of DNA methylation profiles of the human neutrophil.

*A. phagocytophilum* infection induces widespread hypermethylation of the neutrophil genome within 24 h of intracellular infection. Hypermethylation of the neutrophil genome benefits the bacterium and allows for increased colonization of the host cell since inhibition of DNMTs by 5-azacytidine resulted in decreased bacterial loads at 24-72 h. This further suggests that even small changes in DNA methylation affect bacterial growth. Previous studies that investigated neutrophil methylation patterns focused on changes induced by myeloid differentiation and regions of hypomethylation [33, 34]. Despite the hypermethylation of the host genome induced by *A. phagocytophilum*, many of the key regions identified as being hypomethylated remained unmethylated yet had reduced expression in infected cells, suggesting a level of regulation beyond DNA methylation in these regions.

Regions of methylation were found to localize within gene bodies and gene-associated DNA within 3 kb of the transcriptional start and termination sites. Intragenic DNA methylation is associated with regulation of alternative promoters, alternative splicing, and non-coding RNAs (ncRNAs) [38–42]. Genes with intragenic DNA methylation continue to be expressed or have increased expression. It is thought that CpG methylation in intragenic regions results in decreased transcription from alternate promoters, and thus, decreased transcription of regulating ncRNAs that in turn promotes increased target gene transcription [39, 42]. In contrast, Jjingo et al. suggest that increased transcription and gene body methylation could be a result of DNA accessibility to methylating complexes, rather than regulation by ncRNA or expression of alternate promoters [40]. Previously published reports regarding neutrophil transcription in response to *A. phagocytophilum* infection show that more genes are upregulated than downregulated [9–11], and this observation is borne out by the demonstration that among neutrophil genes with newly methylated DNA after *A. phagocytophilum* infection, more with the most significant differential transcription are upregulated than downregulated; however, a demonstration of which genomic features, when DNA hypermethylated, influence differential gene expression could not be deduced by this approach. The chromosome 6 MHC locus was one of the regions with the most differentially expressed genes at 24 h in these datasets, and these findings correlate with new regions of new DNA methylation. This discovery, in light of genome-wide hypermethylation, further supports the hypothesis that epigenetic alterations coordinate infected granulocyte transcriptional reprogramming. Whether the result of ncRNAs, the effects of methylating complexes, or other mechanisms, hypermethylation of intragenic sequences could support marked changes in transcriptional programs, including those that benefit *A. phagocytophilum* survival in neutrophils. Owing to the suboptimal infection levels obtained in the Borjesson et al. studies, the proximate cause of these changes will require further investigation [28].

Closer examination of new methylation sites in infected samples shows higher than expected localization within exons and intron/exon junctions. Rönnerblad et al. independently confirmed a large number of differentially methylated regions in human neutrophils localized after the first exon in gene bodies [33]. DNA methylation at intron/exon junctions is associated with increased H3K36 trimethylation resulting in alternative splice variants [38]. Currently it is not known whether DNA methylation is the precipitating factor for histone H3K36 methylation or vice versa. Given these findings, we hypothesize that deep RNA-seq of *A. phagocytophilum*-infected neutrophils could identify splice variants of highly expressed genes. The localization of newly methylated regions in gene bodies suggests that transcription of these genes could be enhanced and that splice variants are likely to exist; these observations could shed light on some aspects of the transcriptional reprogramming that is key to *A. phagocytophilum* survival and transmission. Previous reports utilizing transcriptional profiling by microarrays [9, 10] are unlikely to detect or quantify the majority of potential variants.

Despite the marked increase in gene-associated DNA methylation induced by *A. phagocytophilum*, there is not a strong genome-wide correlation between infection-induced alterations in gene transcription and annotated regions of meDNA, especially at individual gene or DNA methylation marks. Intragenic DNA methylation seems to have no effect on expression on a genome-wide scale. This could suggest that most newly methylated regions were not transcriptionally active and that the potential for upregulated expression is abrogated by *A. phagocytophilum* infection. We now have evidence to suggest that intergenic DNA methylation influences transcriptional programs of nearby genes as observed across the genome as a whole. For example, within the MHC locus on chromosome 6, regions significantly enriched for meDNA appear to demarcate boundaries of chromatin with increased expression and localize within regions of differential expression. This particular arrangement suggests that DNA methylation, and potentially histone marks, co-contribute to alterations over large chromosomal territories that influence expression of surrounding genes and accompanying transcriptional programs. Based on these observations, DNA methylation or factors that influence DNA methylation and DGE across large genomic regions could be important aspects of *A. phagocytophilum* neutrophil infection, and thus, disease pathogenesis.

The conceptual framework regarding genomic regions of differential methylation has scarcely been examined. However, recent studies in colonic carcinoma reveal that focal regions, >100 kb in size, correlate with regions of attachment to the nuclear lamina, suggesting that factors that bring together distant coordinately regulated genomic regions, such as those tethered by matrix attachment region binding proteins, could guide DNA methylation, histone acetylation, and perhaps ncRNA expression to direct transcriptional reprogramming and pathogen survival [30]. Much remains to be determined, and an important question includes whether *A. phagocytophilum* plays a passive or active role in the process. A compelling hypothesis could revolve around the known translocation of the *A. phagocytophilum* nucleomodulin AnkA that binds to DNA in multiple genomic locations and recruits histone-modifying complexes including those known to interact with DNA methyltransferases and methylated CpG-binding domain proteins [17, 18]. The specificity of AnkA-DNA binding could then dictate genome-wide epigenetic alterations that reprogram cells to support *A. phagocytophilum* survival instead of microbial killing. Indeed, whether such reprogramming events occur as a result of other prokaryotic nucleomodulins is an area of intense research by investigators of microbial pathogenesis. The epigenetic research tools pioneered by oncologists, stem cell biologists, and those who study reprogramming of somatic cells now find applications in microbial pathogenesis studies as well. With mounting evidence of the impact of nucleomodulins from *Shigella*, *Listeria*, *Legionella*, *Chlamydia*, *Mycobacterium*, *Toxoplasma*, and other pathogenic microbes comes the realization that their impact surmounts the classical appreciation of influences over signaling pathways and intracellular trafficking, to the control of entire cellular programs.

## Conclusions

Within 24 h of infection, *A. phagocytophilum* induces hypermethylation of neutrophil DNA on a genome-wide scale. Hypermethylation of the genome is associated with enhanced bacterial growth and colonization of the host cell. DNA methylation was predominantly associated with gene bodies or within 3 kb of transcriptional start and termination sites and tended to localize to exons and intron/exon junctions. Intragenic DNA methylation did not correlate strongly with *A. phagocytophilum*-induced differential gene expression over all chromosomes. However, intergenic DNA methylation is associated with infection-related transcriptional alterations. Targeted investigation of the MHC locus on chromosome 6 demonstrated that newly methylated sites fall within inactive chromatin domains and line the boundaries of active chromatin and that DNA methylation is associated with differential gene expression. Together, these data suggest that *A. phagocytophilum*-induced hypermethylation of the neutrophil genome is important for initial survival and propagation and that its influence over cellular epigenetics can lead to cellular reprogramming of even terminally-differentiated neutrophils.

## Methods

### Cell lines and cell culture

Primary peripheral blood neutrophils were isolated from venous blood of three healthy adult donors (two females, one male) as approved by the Johns Hopkins Medicine IRB and as previously described [43]. Briefly, EDTA-anticoagulated blood was dextran-sedimented and leukocyte-rich plasma centrifuged through a Ficoll-Paque gradient. Mononuclear cells were removed and the remaining erythrocytes were lysed in hypotonic saline. HL-60 promyelocytic cells (ATCC CCL-240) were purchased from American Type Tissue Culture (Manassas, VA, USA). Both neutrophils and HL-60 cells were maintained or propagated in RMPI 1640 (Hyclone, Thermo Fisher Scientific, Waltham, MA, USA) with 10 % fetal bovine serum (Thermo Fisher Scientific) and Glutamax (Life Technologies, Carlsbad, CA, USA). HL-60 cells were differentiated for 3 to 5 days prior to infection with 1 μM all-*trans* retinoic acid (ATRA). All cells were grown at 37 °C in a humidified incubator with 5 % CO_2_. Where stated, HL-60 cells were treated with 5-azacytidine (Sigma-Aldrich, St. Louis, MO, USA) in DMSO for 24 h and separately with RG108 (Sigma-Aldrich, St. Louis, MO, USA) in DMSO for 24, 48, and 72 h, or with DMSO alone as a vehicle control.

### *Anaplasma phagocytophilum* propagation, neutrophil infection, and quantitation

*A. phagocytophilum* was maintained in HL-60 cells as previously described [44]. Cell-free bacteria were obtained from HL-60 cells of which 90–95 % were infected. Infected cells were centrifuged at 500× g for 5 min and resuspended in 1× PBS. Infected cells were lysed by sonication using an output of four of a Branson Sonifier 250 (Branson Ultrasonics, Danbury, CT, USA) for 15 s or via syringe lysis using a 22 g syringe. Lysed cells were then centrifuged at 1000× g for 10 min, and the bacteria-enriched supernatant was centrifuged at 13,000× g for 30 min. The bacterial pellets were suspended in RMPI 1640 medium.

Neutrophils were infected ex vivo in triplicate using a multiplicity of infection (MOI) of 25:1 and 100:1 for experiments using HL-60 cells. An estimate of 10 infectious bacteria per infected HL-60 cell was used. The proportion of infected cells was determined at 24 h after Romanowsky staining (Protocol HEMA3, Thermo Fisher Scientific) of cytocentrifuged cells. In addition and for experiments to test the effect of DNA methyltransferase inhibitors on *A. phagocytophilum* propagation, DNA extracted from these cultures (DNeasy Blood and Tissue Kit, Qiagen, Valencia, CA, USA) was used in a quantitative real time 5′ nuclease PCR assay that targets *A. phagocytophilum msp2/p44* normalized to human *ACTB* [16]. A standard curve of cloned *msp2* was used to accurately estimate the number of bacteria per cell, providing that the *A. phagocytophilum* genome encodes approximately 100 *msp2/p44* genes [45]. For experiments that examined the effects of DNA methyltransferase inhibition on *A. phagocytophilum* growth, 8-azacytosine and RG108 were initially diluted in DMSO, then diluted to working concentrations (200, 100, and 50 nM for 8-azacytosine and 100, 50, and 25 μg/mL for RG-108) in RPMI 1640 medium. DMSO diluted to equivalent concentrations as used in the 200 nM 8-azacytosine or 100 μg/mL RG-108 preparations was used as vehicle controls.

### MBD-Seq library preparation

Enrichment of 5-methylcytosine modified DNA was performed using DNA extracted from ex vivo infected and uninfected neutrophils from there healthy donor subjects 24 h post ex vivo infection. DNA libraries isolated by MBD enrichment were analyzed by MBD-seq to determine sites of MBD protein binding and DNA methylation (SKCCC Next Generation Sequencing Core). MBD-seq was carried out as described previously [46]. Briefly, 2 μg of DNA was sonicated to an average molecular size of ~150–250 bp and end-repaired using the NEBNext SOLiD DNA library preparation kit end-repair module following the manufacturer’s protocol (New England Biolabs, Ipswich, MA, USA). Fragments were purified using a Qiagen PCR purification kit (Qiagen). SOLiD P1 and P2 adapters lacking 5′ phosphate groups (Life Technologies) were ligated to the fragment using the NEBNext adapter ligation module, column-purified, and subjected to isothermal nick-translation by treating with Platinum Taq polymerase to remove the nick. The resulting library was divided into two fractions, a total input fraction, and an enriched methylated fraction. The enriched methylated fraction was subjected to affinity enrichment of methylated DNA fragments using recombinant C-terminal 6xHis-tagged MBD2-MBD polypeptides immobilized on magnetic beads, similar to previously described methods [47–49]. The resulting enriched methylated fraction and the total input fraction were then subjected to library amplification using the NEBNext amplification module according to the manufacturer’s protocols, using 4–6 cycles for the total input, and 10–12 cycles for the enriched methylated fractions. Library fragments between 200–300 bp were selected after agarose gel electrophoresis. The libraries were then subjected to emulsion PCR and bead enrichment following the SOLiD emulsion PCR protocol (Life Technologies). The resulting beads were then deposited on the SOLiD flow cell and subjected to massively parallel 50 bp single-read sequencing on a Life Technologies SOLiD sequencer. Reads were mapped to the human genome hg18 build using Bioscope software. The total number of reads per sample and the percentage alignment are summarized in Additional file 3: Table S1. Regions of methylated DNA enrichment were identified using MACS [25] and SICER [26] as described previously [50]. Additional file 4: Table S2 summarizes the number of peaks called with both MACS and SICER as well as delineates the total number of peaks above various thresholds of −10*log_10_ (*p* value) >50, >100, and >200. These and all subsequent DNA methylation studies were conducted independently using the three individual donors’ neutrophils, and all data is shown as a single donor representative of all three, or as an average of all three donors’ results, as noted in the figures.

### Luminometric Methylation Assay and 5-methylcytosine DNA ELISA

LUMA was performed by EpigenDx (Hopkinton, MA, USA) as described by Karimi et al. [51]. Briefly, samples were digested with *Hpa*II + *Eco*RI or *Msp*I + *Eco*RI, and fragments were amplified by Pyrosequencing™ (Qiagen). The ratio of (dGTP + dCTP)/dATP was calculated for *Hpa*II*/Eco*RI and *Msp*I*/Eco*RI fragments, and the percentage of methylation was determined by the ratio of (*Hpa*II*/Eco*RI)/(*Msp*I*/Eco*RI). Samples were tested in duplicate. 5-Methylcytosine was quantified in DNA preparations from RG108-treated cultures at days 0–3 postinfection using a 5-mC DNA ELISA (Zymo Research Corp., Irvine, CA, USA). The quantity of 5-methyl cytosine DNA was quantified by comparison with a standard curve and normalized to the content of day 0 no drug control cultures.

### Identifying gene regions enriched for DNA methylation

Publically available cis-regulatory element annotation (CEAS) software [52, 53] was used to characterize regions of methylated DNA enrichment by calculating the fraction present in different gene regions (introns, exons, 5′ UTR, 3′UTR, and distal intergenic regions), promoters, bi-directional promoters, and regions downstream of gene bodies; this was also used to create average profile analyses of the smoothed adjusted log_2_ (M/T) values across transcriptional start sites (TSS), transcriptional termination sites (TTS), long (2715–11,673 bp), medium (842–2715 bp), and short (158–842 bp) intronic sequences and long (164–483 bp), medium (109–164 bp), and short (66–109 bp) exonic sequences.

### Identifying chromosomal locations enriched for DNA methylation and differential gene expression

Publically available transcription microarray data from *A. phagocytophilum*-infected neutrophils was re-analyzed using RMA [9]. Only the 24 h time point was used for comparison with the same interval in the DNA methylation studies. Differential expression was determined based on the standard deviation of the fold-change from the mean. Infected and uninfected fold-change gene expression data were transformed to log_2_ for all analyses.

Regions enriched for DNA methylation and differential expression were organized linearly among their respective chromosomes and divided into bins of ~one million base pairs. The number of gene features (expression) and methylation peaks within each bin were counted and compared to the average number across the genome to determine locations of interest. Regions of interest were defined by those bins which had significantly more counts than average for that chromosome.

### DNA methylation and transcription in *A. phagocytophilum*-infected neutrophils

Because *A. phagocytophilum* infection-induced genome-wide DNA hypermethylation, we investigated whether this affected gene transcription. Borjesson et al. previously investigated the effect of *A. phagocytophilum* infection on transcription of the neutrophil using microarrays [9]. To access and compare these data to the DNA methylation analyses, the transcriptional profiling data were obtained from Gene Expression Omnibus (GEO accession number GSE2405) and re-normalized using RMA, as described above. Differential expression was determined for 24 h postinfection, and genes were sorted by chromosome and physical location based on annotations in human genome assembly hg18 used for both these data and the DNA methylation studies. To account for underestimations of significant discoveries using *p* values, we calculated the FDR adjusted *p* values, or *q* values, for each Spearman correlation between windows of DNA methylation and windows of differential gene expression on chromosome 6. We selected a FDR of a maximum of 10 % as a significant correlation.

## Additional files

Additional file 1: Figure S1. Treatment of ATRA-differentiated HL-60 cells infected with *A. phagocytophilum* with DNMT inhibitor RG108 reduces 5-methyl cytosine genomic content. The proportion of 5-methyl cytosine in drug-treated cultures vs. no drug on day 0 results is reduced at days 1–3 for most doses, and at all days for the 100 μg/mL dose, suggesting that the main target of the inhibitor is DNA methylation as an explanation for reduced microbial growth. Additional file 2: Figure S2. Intragenic and intergenic DNA methylation correlate with differential gene expression within the MHC locus and downstream region. Increased DNA methylation within intragenic regions across the MHC locus correlates with differential gene expression. Conversely, intergenic DNA methylation downstream (PROX) of the MHC locus is correlated with differential gene expression. MeDNA data are from the average of 3 donors. Expression data are derived from GSE2405 [9]. Additional file 3: Table S1. Genome coverage. Genome coverage as dictated by the total number of reads for enriched and total input fractions. The % alignment was calculated as the percentage of reads that aligned to the hg18 human reference genome. Data from all 3 donors is shown. Additional file 4: Table S2. Number of peaks called using increasing stringency. Significant peaks were called using MACS v1.4 and SICERV1.1. Significant peaks were sorted by the −10*log10(*p* value) score based on increasing stringencies to determine the number of peaks present above thresholds of >50, >100 and >200. Data from all 3 donors is shown.

## Abbreviations

ρ: Spearman correlation coefficient (rho)
ACTB: beta-actin
ATRA: all-transretinoic acid
BCL2: B-cell lymphoma 2 gene
CDKN2A: cyclin-dependent kinase inhibitor
CDS: coding sequences
CEAS: cis-regulatory element annotation software
ChIP: chromatin immunoprecipitation
CpG: C-phosphate-G
CYBB: cytochrome b-245 beta chain gene
DGE: differential gene expression
DMSO: dimethyl sulfoxide
DNMT: DNA methyltransferase
EDTA: ethylenediaminetetraacetic acid
EMZL: extranodal marginal zone B-cell lymphoma
GEO: Gene Expression Omnibus
gp91phox: heme-binding subunit of NADPH oxidase
H3K26: histone 3 lysine 26
HDAC: histone deacetylase
HL-60: a human promyelocytic cell line
IL-1α: interleukin-1 alpha
IL-8: interleukin-8
IQR: interquartile range
LUMA: Luminometric Methylation Assay
MACS: model-based analysis of ChIP-Seq software
MBD: DNA methyl binding protein
MBD-seq: methylated DNA binding domain protein immunoprecipitation of bound DNA followed by sequence analysis
meDNA: methylated DNA
MHC: major histocompatibility complex
NADPH: nicotinamide adenine dinucleotide phosphate
ncRNA: non-coding RNA
PROX: genomic region downstream of MHC locus on chromosome 6
RAC2: ras-related C3 botulinum toxin substrate 2 gene
RMA: robust multi-array average normalization
SICER.df: Statistical model for Identification of ChIP-Enriched Regions software
TSS: transcriptional start site
TTS: transcription termination site
UTR: untranslated region
